# Melanoma risk loci as determinants of melanoma recurrence and survival

**DOI:** 10.1186/1479-5876-11-279

**Published:** 2013-11-04

**Authors:** Justin Rendleman, Shulian Shang, Christine Dominianni, Jerry F Shields, Patrick Scanlon, Christina Adaniel, Alexis Desrichard, Michelle Ma, Richard Shapiro, Russell Berman, Anna Pavlick, David Polsky, Yongzhao Shao, Iman Osman, Tomas Kirchhoff

**Affiliations:** 1 New York University Cancer Institute, New York University School of Medicine, New York, NY 10016, USA; 2 Division of Epidemiology, New York University School of Medicine, New York, NY, USA; 3 Division of Biostatistics, New York University School of Medicine, New York, NY, USA; 4 Ronald O. Perelman Department of Dermatology, New York University School of Medicine, New York, NY, USA; 5 Interdisciplinary Melanoma Cooperative Group, New York University School of Medicine, New York, NY, USA; 6 Department of Surgery, New York University School of Medicine, New York, NY, USA; 7 Department of Medicine, New York University School of Medicine, New York, NY, USA

## Abstract

**Background:**

Steadily high melanoma mortality rates urge for the availability of novel biomarkers with a more personalized ability to predict melanoma clinical outcomes. Germline risk variants are promising candidates for this purpose; however, their prognostic potential in melanoma has never been systematically tested.

**Methods:**

We examined the effect of 108 melanoma susceptibility single nucleotide polymorphisms (SNPs), associated in recent GWAS with melanoma and melanoma-related phenotypes, on recurrence-free survival (RFS) and overall survival (OS), in 891 prospectively accrued melanoma patients. Cox proportional hazards models (Cox PH) were used to test the associations between 108 melanoma risk SNPs and RFS and OS adjusted by age at diagnosis, gender, tumor stage, histological subtype and other primary tumor characteristics.

**Results:**

We identified significant associations for rs7538876 (*RCC2*) with RFS (HR = 1.48, 95% CI = 1.20-1.83, p = 0.0005) and rs9960018 (*DLGAP1*) with both RFS and OS (HR = 1.43, 95% CI = 1.07-1.91, p = 0.01, HR = 1.52, 95% CI = 1.09-2.12, p = 0.01, respectively) using multivariable Cox PH models. In addition, we developed a logistic regression model that incorporates rs7538876, rs9960018, primary tumor histological type and stage at diagnosis that has an improved discriminatory ability to classify 3-year recurrence (AUC = 82%) compared to histological type and stage alone (AUC = 78%).

**Conclusions:**

We identified associations between melanoma risk variants and melanoma outcomes. The significant associations observed for rs7538876 and rs9960018 suggest a biological implication of these loci in melanoma progression. The observed predictive patterns of associated variants with clinical end-points suggest for the first time the potential for utilization of genetic risk markers in melanoma prognostication.

## Introduction

Cutaneous melanoma (CM) is one of the few cancers which have displayed an increasing incidence and, more importantly, a steady mortality rate over the past decade [1,2]. While the increase of CM incidence has partially been attributed to more proficient clinical screening techniques [2-4], there has been little improvement in the ability to accurately assess patient prognosis at the time of diagnosis. This is particularly apparent in the difficulties of predicting recurrent/metastatic disease among early-stage melanoma patients; while the 5-year survival rate for localized melanoma is >99%, that of regional and distant metastasis dramatically decreases to 65.8% and 15.2%, respectively [2]. Due to the disease heterogeneity and limited specificity, current clinicopathological variables used in the prognostication and staging of melanoma, as defined by the American Joint Committee on Cancer (AJCC) [5,6], are not sufficient for a more personalized clinical assessment [7,8]. This urges for the development of complementary biomarkers with specific prognostic potential allowing for more focused clinical surveillance of CM patients with increased risk of developing recurrent and/or metastatic disease [8-10]. Germline genetic markers have been proposed to provide individualized utility in melanoma prognosis [11-18]. However, the limited selection of candidate variants and insufficient study power were among the main factors complicating the accurate estimates of clinical end-points associated with the genetic variants in these prior studies.

Genome-wide association analyses (GWAS) have recently identified a myriad of genetic loci associated with the risk of melanoma and/or melanoma host-related phenotypes, such as pigmentation or tanning response. While in several common cancer models we and others have shown that the risk loci, including those from recent GWAS, may represent novel biomarkers of clinical outcomes [19-21], in melanoma the impact of genetic risk markers on disease progression was never systematically tested. To evaluate the prognostic potential of melanoma germline genetic risk loci, in the current study we have examined the correlation between the clinical outcomes of 891 melanoma patients and 108 common variants previously shown to be associated with risk of melanoma and melanoma related phenotypes in recent GWAS. To the best of our knowledge this is to date the most comprehensive assessment of common genetic risk variants for their use as novel biomarkers of melanoma prognosis.

## Methods

### Study population

960 patients (Table 1) receiving treatment for primary melanoma at New York University (NYU) Langone Medical Center were prospectively enrolled in the Interdisciplinary Melanoma Cooperative Group (IMCG) database from August 2002 to December 2011 [22]. The study was approved by the Internal Review Board (IRB) of NYU, and all patients signed informed consent at time of enrollment. For each patient, DNA specimens (extracted from blood) and prospective clinical and pathological data were collected. This also included basic demographic information such as age at diagnosis, sex, and ethnicity. Ethnicity was determined based on self-reported ancestry; the majority of patients in the study were of white Caucasian ethnicity, including a subset of Ashkenazi Jewish (AJ) ancestry (n = 204, 22%). A small fraction of patients were of other non-Caucasian ethnicities (n = 35, 3.5%). The clinical data in this study included 2009 AJCC stage at pathological diagnosis, sentinel lymph node (SLN) status, and the primary tumor characteristics including thickness, ulceration status, mitotic rate, anatomic site, and histological type.

**Table 1:** Study population statistics summarizing patient and primary tumor characteristics

**Age at pathological diagnosis (years)**		**Primary tumor thickness (mm)**	
Median (Range)	58 (15–97)	Median (Range)	0.97 (0.1-33)
**Self-reported ethnicity**		**AJCC**^a^**stage at pathological diagnosis**	
Ashkenazi Jewish	204 (22.9%)	I	566 (63.5%)
Irish	95 (10.7%)	II	150 (16.8%)
Italian	46 (5.2%)	III	150 (16.8%)
Other non-hispanic white	511 (57.3%)	IV	23 (2.6%)
Other	35 (3.9%)	Unclassified	2 (0.2%)
**Gender**		**Primary tumor ulceration**	
Male	501 (56.2%)	Absent	684 (76.8%)
Female	390 (43.8%)	Present	158 (17.7%)
		Unclassified/Unknown	49 (5.5%)
**Family history of melanoma**		**Primary tumor mitosis**	
No	727 (81.6%)	Absent	326 (36.6%)
Yes	139 (15.6%)	Present	479 (53.8%)
Unknown	25 (2.8%)	Unclassified/Unknown	86 (9.6%)
**Sentinel lymph node positive**		**Primary tumor anatomic site**	
No	774 (86.9%)	Axial	469 (52.6%)
Yes	117 (13.1%)	Extremity	378 (42.4%)
		Unclassified/Unknown	44 (4.9%)
**Status at last follow-up**		**Primary tumor histological subtype**	
Alive, no melanoma	685 (76.9%)	Superficial spreading melanoma	470 (52.7%)
Alive, with melanoma	31 (3.5%)	Nodular melanoma	223 (25.0%)
Alive, status unknown	28 (3.1%)	Acral lentiginous melanoma	25 (2.8%)
Died, no melanoma	14 (1.6%)	Lentigo maligna melanoma	24 (2.7%)
Died, with melanoma	131 (14.7%)	Desmoplastic melanoma	31 (3.5%)
Died, status unknown	2 (0.2%)	Other melanoma	37 (4.2%)
		Unclassified/Unknown	81 (9.1%)
**Recurrence**		**Multiple primary melanoma**	
No	639 (71.7%)	No	745 (83.6%)
Yes	252 (28.3%)	Yes	144 (16.2%)
		Unknown	2 (0.2%)

^a^AJCC, American Joint Committee on Cancer.

### Selection of single nucleotide variants and genotyping

A total of 139 genetic variants were selected through the comprehensive search of published data from GWAS on melanoma risk, nevi-driven phenotypes, pigmentation, hair color, skin color and other melanoma risk etiologies. The selection criteria focused on variants with the most significant associations reported from each of these published GWAS. While selection priority was given to SNPs that achieved genome-wide level of significance in at least one of these studies (p < 10^-7^), we have also included other top SNPs from these scans that did not reach genome-wide level of significance, but map in the regions of the most significant associations (see p-values and respective references in Additional file 1). Genotyping of 139 selected variants was performed using the highly multiplexed Sequenom MassARRAY system (Sequenom Inc., CA). Quality control (QC) measures included duplicates (8 per each 384-well plate) and non-template controls (2 per plate) resulting in >99% observed concordance with no evidence of cross-contamination. Post-genotyping filtering included the following criteria: exclusion of SNPs with minor allele frequency (MAF) <5%, exclusion of SNPs with a call rate <95%, exclusion of samples with a call rate <95%, and exclusion of SNPs with significant departure from Hardy Weinberg equilibrium (p < 0.001). The resulting filtered data contained the genotype information of 108 variants for 891 melanoma patients.

### Statistical analysis

Cox proportional hazards models (Cox PH) were used to assess the associations between each SNP and recurrence-free survival (RFS) and overall survival (OS). SNP associations were analyzed under both a co-dominant model (2-degree freedom; 2df test) and an additive model. Multivariable analyses were stratified by tumor stage and adjusted by clinicopathological covariates: age and thickness as continuous covariates; gender, ulceration status (present/absent), and anatomic site (axial/extremity) as dichotomous covariates; and histological type as categorical covariates. Because of the AJ ancestry present in our population (n = 204, 22%), all analyses were also corrected for possible population stratification by adjustment for AJ status. Time at risk was calculated from the date of diagnosis to the date of event (RFS-recurrence, OS-death) or date of last follow up. Cox proportional hazard models were also used for subgroup analyses for tumor thickness, ulceration, anatomic site, and histological type (superficial spreading melanoma -SSM and nodular melanoma -NM), leaving out the sub-grouping variable (thickness, ulceration, anatomic site or histological type) from the adjustment covariates in each respective subgroup analysis. Associations between SNPs and clinical covariates were also tested using logistic regression (for ulceration status and anatomic site) and linear regression (for tumor thickness) analyses, with adjustments for age, gender, and ethnicity. To test the predictive utility of candidate SNPs in a model inclusive of clinical covariates, logistic regression was fitted with 3-year recurrence (yes/no) as a response. Receiver Operating Characteristic (ROC) curves were constructed from the logistic regression model and the area under the ROC curve was used to assess the classification performance of the model. The statistical significance of area under curve (AUC) change was assessed by DeLong’s test [23]. All statistical analyses were conducted using R 2.12.0. For all analyses we have also controlled for multiple testing by applying Bonferroni correction. Out of the 108 SNPs tested in the study, 64 SNPs passed an independence threshold with Pearson’s correlation coefficient (r-square) <0.6. We therefore have determined the number of independent tests as 64 and thus define the Bonferroni adjusted significance level in this study as 0.05/64, considering the significant p-value after Bonferroni correction as p < 0.0008.

The SNP-gene and SNP-CpG associations were tested by incorporating expression (expression quantitative trait loci – eQTL) and methylation (methylation quantitative trait loci -meQTL) information assessed by Genevar [24] on adipose tissue from a population of 428 female twin-pairs (856 individuals), collected as a part of the Multiple Tissue Human Expression Resource (MuTHER) [25], combining Illumina 610 k or 1 M chip, Illumina HT-12v3 expression arrays with methylation data from 27 k Illumina array. The eQTL/meQTL associations were calculated by Spearman’s rank correlation tests.

The variants with high correlation (proxies) with the top associated SNPs were identified by querying the most recent data of 1000 Genomes Project (1KGP), by standard Pearson’s correlation coefficient (r-square) >0.90. The identified proxies were assessed for functional impact by ANNOVAR [26], implementing the data from the Encyclopedia of DNA Elements (ENCODE) [27], focusing on 8 functional categories: coding regions, conserved transcription factor (TF) binding sites, TF binding sites based on ChIP-Seq data (using ENCODE database), enhancer sites based on H3K4me1 chromatin marks (using ENCODE database), DNase I hypersensitivity clusters (using ENCODE database), known CNVs, and 3′ UTR, and 5′ UTR.

## Results

In this study, 960 melanoma samples have been genotyped for 139 SNPs, associated in recent GWAS with melanoma risk and other melanoma-related phenotypes (Additional file 1). Patient demographic and clinicopathological characteristics of the study population are summarized in Table 1. After applying quality control filters (see Methods), we have collected the genotype data from 108 SNPs in 891 melanoma patients to be used for the association analysis.

**Figure 1:**
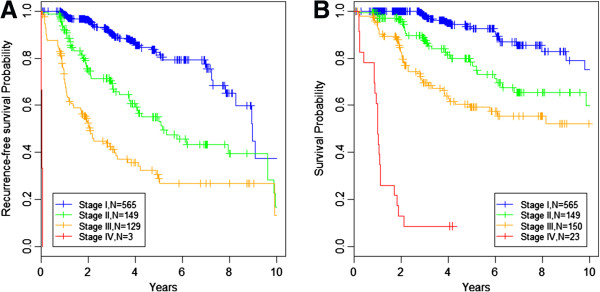
**Kaplan-Meier curves for recurrence-free and overall survival stratified by stage at pathological diagnosis. A)** Kaplan-Meier curves plotting recurrence-free survival probability against time, stratified by stage at diagnosis. Stage I blue line, n = 565. Stage II green line, n = 149. Stage III orange line, n = 129. Stage IV red line, n = 3. **B)** Kaplan-Meier curves plotting overall survival probabilities against time, stratified by stage at diagnosis. Stage I blue line, n = 565. Stage II green line, n = 149. Stage III orange line, n = 150. Stage IV red line, n = 23.

From the univariate analysis of clinicopathological variables using Cox PH model, 8 clinical covariates were found to be significantly associated with RFS and OS (Table 2). These included pathological stage at diagnosis (Kaplan Meier curves in Figure 1), SLN positivity, age at diagnosis, and the primary tumor characteristics: thickness, ulceration status, histological type, anatomic site, and mitotic index.

**Table 2:** Summary of clinicopathological associations with recurrence-free and overall survival

**Variable**		**Recurrence-free survival**		**Overall survival**				
	**HR**	***P***	**HR**	***P***				
**Stage**				
I	Ref		Ref	
II	3.06	1.0x10^-9^	3.19	1.2x10^-06^
III	8.00	<2x10^-16^	5.07	2.0x10^-13^
IV	192.50	<2x10^-16^	52.8	<2x10^-16^
**Gender**				
Female	Ref		Ref	
Male	1.25	0.093	1.24	0.21
**Self-reported ethnicity**				
Non-AJ	Ref		Ref	
AJ	0.85	0.29	0.72	0.13
**Sentinel lymph node status**				
Negative	Ref		Ref	
Positive	3.29	<2x10^-16^	2.83	1.1x10^-08^
**Family history of melanoma**				
No	Ref		Ref	
Yes	0.86	0.4	0.66	0.11
**Primary tumor ulceration**				
Absent	Ref		Ref	
Present	3.75	<2x10^-16^	3.13	5.8x10^-10^
**Primary tumor mitotic index**				
None	Ref		Ref	
Few	2.13	0.002	1.63	0.092
Moderate	4.27	5.4x10^-10^	5.11	2.8x10^-09^
Many	7.23	<2x10^-16^	2.36	0.004
**Primary tumor histological type**				
SSM	Ref		Ref	
ALM	6.60	4.6x10^-11^	6.6	9.1x10^-08^
DM	3.42	0.0004	2.36	0.056
LMM	2.10	0.12	3.4	0.022
NM	4.48	<2x10^-16^	3.68	4.4x10^-09^
Other	1.44	0.37	1.72	0.31
**Primary tumor anatomic site**				
Axial	Ref		Ref	
Extremity	0.72	0.026	0.66	0.033
**Age at pathological diagnosis**				
	1.01	0.035	1.02	1.20x10^-05^
**Primary tumor thickness (mm)**				
	1.11	<2x10^-16^	1.1	5.30x10^-11^

To test the associations of 108 melanoma risk variants with RFS and OS, we first used a univariate Cox PH analysis. The most significant associations with RFS were observed for rs966321 on chromosome 1p36.32 (HR = 0.79, additive p = 0.007), rs154659 on chromosome 16 near *MC1R* (HR = 1.29, additive p = 0.009) and rs6088520 at 20q11.22 (HR = 0.78, additive p = 0.006) (Table 3A). While rs6088520 is a melanoma risk allele, rs966321 and rs154659 were originally identified in a GWAS on tanning phenotypes. Additional borderline associations with RFS in the univariate analysis included rs7538876 near *RCC2* and another SNP near *MC1R,* rs7188458, in low linkage disequilibrium (LD) with rs154659 (r^2^ < 0.2). Significant univariate associations with OS included rs10861741 (HR = 0.59, additive p = 0.008) in *BTBD11*, previously shown to be associated with hair color in European ancestries, and rs9960018 (HR = 1.47, additive p = 0.009) in *DLGAP1* on chromosome 18p11.31, previously linked with tanning response.

**Table 3:** Summary of SNP associations with recurrence-free and overall survival

**A. Survival association results from genetic univariate Cox proportional hazards models.**											
**SNP**	**Locus**	**Gene**	**prior GWAS associations**	**MAF**	**Genotype**	**Recurrence-free survival**			**Overall survival**		
						**HR**	**95% CI**	***P***	**HR**	**95% CI**	***P***
rs966321	1p36.32	-	Tanning; β = -.14, p = 1e-9	0.49	AA	Ref			Ref		
					CA	0.82	0.62-1.08		0.76	0.52-1.09	
					CC	0.61	0.43-0.88	0.02	0.71	0.45-1.12	0.23
					Additive	0.79	0.66-0.94	0.007	0.83	0.66-1.05	0.11
rs7538876	1p36.13	Near RCC2	BCC; OR = 1.28, p = 4e-12	0.32	GG	Ref			Ref		
					AG	1.09	0.83-1.43		0.97	0.68-1.38	
					AA	1.69	1.18-2.43	0.02	1.09	0.67-1.77	0.89
					Additive	1.25	1.04-1.50	0.01	1.03	0.81-1.29	0.83
rs10861741	12q23.3	BTBD11	Hair; β = .12, p = 1e-4	0.15	CC	Ref			Ref		
					TC	0.91	0.68-1.21		0.60	0.39-0.91	
					TT	0.13	0.02-0.94	0.01	0.31	0.04-2.20	0.01
					Additive	0.78	0.60-1.01	0.06	0.59	0.40-0.87	0.008
rs154659	16q24.3	Near MC1R	Tanning; β = .14, p = 7e-8	0.28	TT	Ref			Ref		
					CT	1.18	0.90-1.53		0.97	0.69-1.37	
					CC	1.84	1.21-2.79	0.02	1.14	0.63-2.05	0.88
					Additive	1.29	1.06-1.56	0.009	1.03	0.80-1.32	0.84
rs7188458	16q24.3	Near MC1R	CM; OR = 1.3, p = 1e-12	0.44	GG	Ref			Ref		
			Tanning; β = .13, p = 8e-7		AG	1.54	1.13-2.10		1.43	0.95-2.15	
			Hair; β = .16, p = 4e-12		AA	1.29	0.88-1.89	0.01	1.04	0.62-1.75	0.12
					Additive	1.15	0.96-1.37	0.12	1.04	0.82-1.31	0.76
rs9960018	18p11.31	DLGAP1	Tanning; β = -.15, p = 1e-5	0.12	CC	Ref			Ref		
					TC	1.29	0.96-1.75		1.63	1.13-2.36	
					TT	1.83	0.90-3.72	0.09	1.65	0.67-4.06	0.03
					Additive	1.32	1.03-1.68	0.02	1.47	1.10-1.96	0.009
rs6088520	20q11.22	Near	CM; OR = .86, p = .02	0.49	CC	Ref			Ref		
		MAP1LC3A			TC	0.80	0.60-1.07		0.92	0.63-1.34	
					TT	0.61	0.42-0.87	0.02	0.68	0.42-1.09	0.22
					Additive	0.78	0.65-0.93	0.006	0.83	0.66-1.04	0.10
**B. Survival association results from multivariable Cox proportional hazards models.**											
rs7538876	1p36.13	Near RCC2	BCC; OR = 1.28, p = 4e-12	0.32	GG	Ref			Ref		
					AG	1.25	0.90-1.72		1.38	0.68-2.83	
					AA	2.41	1.58-3.68	**0.0002**†	1.49	0.43-5.19	0.61
					Additive	1.48	1.20-1.83	**0.0005**‡	1.08	0.82-1.41	0.60
rs12913832	15q13.1	HERC2	Tanning; β = -.19, p = 1e-10	0.36	GG	Ref			Ref		
			Hair; β = -.44, p = 9e-78		AG	0.79	0.58-1.09		0.55	0.26-1.15	
			CM; OR = 0.69, p = 4e-8		AA	0.55	0.34-0.86	0.02	0.29	0.09-1.01	0.05
					Additive	0.75	0.61-0.93	0.007	0.75	0.56-0.98	0.03
rs7188458	16q24.3	Near MC1R	CM; OR = 1.3, p = 1e-12	0.44	GG	Ref			Ref		
			Tanning; β = .13, p = 8e-7		AG	1.52	0.97-2.37		1.05	0.45-2.45	
			Hair; β = .16, p = 4e-12		AA	1.85	1.26-2.70	0.005	1.39	0.50-3.81	0.78
					Additive	1.23	1.01-1.52	0.04	1.12	0.84-1.48	0.44
rs7195066	16q24.3	FANCA/	Hair; β = -.11, p = 2e-6	0.27	CC	Ref			Ref		
		Near MC1R			TC	1.47	1.08-2.01		1.71	0.85-3.46	
					TT	1.03	0.60-1.77	0.04	0.46	0.05-3.90	0.16
					Additive	1.16	0.94-1.43	0.17	0.98	0.75-1.30	0.91
rs9960018	18p11.31	DLGAP1	Tanning; β = -.15, p = 1e-5	0.12	CC	Ref			Ref		
					TC	1.17	0.82-1.68		1.59	0.71-3.58	
					TT	3.73	1.76-7.87	0.01	4.86	1.24-19.0	0.09
					Additive	1.43	1.07-1.91	0.01	1.52	1.09-2.12	0.01
rs6088520	20q11.22	Near	CM; OR = .86, p = .02	0.50	CC	Ref			Ref		
		MAP1LC3A			TC	0.75	0.53-1.07		0.59	0.27-1.30	
					TT	0.55	0.35-0.85	0.02	0.58	0.22-1.51	0.36
					Additive	0.74	0.59-0.92	0.007	0.90	0.68-1.19	0.46

A) Results from the univariate Cox proportional hazards model. B) Results from the multivariate Cox proportional hazards model stratified by stage and adjusted by age, gender, ethnicity, tumor thickness, ulceration status, anatomic site, and histological type. Shown in bold are associations remaining significant after Bonferroni correction based on the number of independent tests (n = 64). Associations with melanoma and melanoma related host phenotypes observed in prior GWAS studies are also listed for each SNP.

† Bonferroni adjusted p-value = 0.01. ‡ Bonferroni adjusted p-value = 0.03.

**Figure 2:**
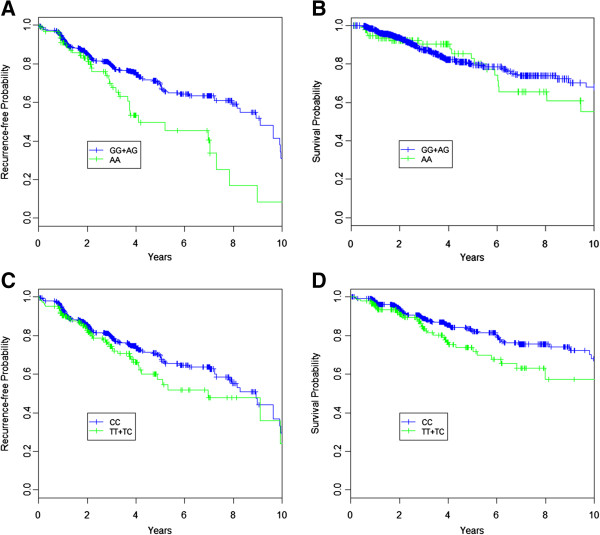
**Kaplan-Meier curves for recurrence-free and overall survival stratified by rs7538876 and rs9960018. A)** Recurrence-free survival probability against time, stratified by rs7538876 assuming a recessive model. **B)** Overall-survival probability against time, stratified by rs7538876 assuming a recessive model. **C)** Recurrence-free survival probability against time, stratified by rs9960018 assuming a dominant model. **D)** Overall-survival probability against time, stratified by rs9960018 assuming a dominant model.

By stratifying for stage and adjusting for 7 clinical covariates, the multivariable analysis identified 6 SNPs significantly associated with RFS (Table 3B). Among these, rs7538876 shows the most significant associations under both 2df test and additive models (p = 0.0002, HR = 2.41, p = 0.0005, HR = 1.48, respectively), passing the Bonferroni correction for multiple testing (adjusted p = 0.01, p = 0.03, respectively). To test whether the observed associations with rs7538876 were confounded by the presence of AJ ancestry in our population, we have also performed sub-analyses separately for AJ and non-AJ patients and saw largely comparable significant effects in both comparisons. Also, in the AJ-unadjusted main effect analysis, no alterations on the effect size or statistical significance were noted compared to the AJ-adjusted results (additive p = 0.0002), indicating that AJ ancestry does not significantly affect the observed association of rs7538876 with RFS in our data. Other associations with RFS were also observed for rs9960018 (homozygous HR = 3.73, 2-df p = 0.0106) and rs7188458 (heterozygous HR = 1.85, 2-df p = 0.0049). For OS, statistically significant associations in multivariable analysis were observed for rs12913832 (HR = 0.75, additive p = 0.0389) in the *HERC2/OCA2* locus on chromosome 15 and rs9960018 (HR = 1.52, additive p = 0.0138). Both rs7538876 and rs9960018 were associated with RFS and OS, respectively across multiple analyses, including multivariable and univariate comparisons. For illustrative purposes, Kaplan Meier curves of the association with RFS and OS for rs7538876 and rs9960018 are shown in Figure 2. As shown in Table 4, we have also found associations of genetic variants with survival outcomes in the subgroups of histological type, ulceration status and tumor thickness. Specifically, among patients with nodular melanoma (NM), rs9960018 and rs12913832 were associated with both RFS and OS. While these SNPs showed associations with RFS and OS in the analyses of all melanoma patients, the associations were stronger among NM patients. Significant associations with OS were also found for rs12750212 and rs1805761 in patients with tumor ulceration (HR = 2.89, p = 0.0023; HR = 1.72, p = 0.0060, respectively). For RFS the most significant associations were observed for rs7538876 (*RCC2*) in patients with superficially spreading melanoma (SSM) (HR = 2.30, p = 0.0002), and rs6088520 in patients with an intermediate tumor thickness (1-4 mm) (HR = 0.61, p = 0.0004). Both of these associations pass Bonferroni correction (adjusted p-values: p = 0.013, p = 0.025, respectively).

**Table 4:** Subgroup multivariate analysis of SNP associations with recurrence-free survival and overall survival using Cox proportional hazards model

**SNP**	**Subgroup**	**Recurrence-free survival**		
		**HR**	**CI 95%**	***P***
rs7538876	Ulceration absent	1.63	1.21-2.10	0.001
	Axial	1.47	1.13-1.91	0.003
	Extremity	1.86	1.28-2.70	0.001
	Thickness <1 mm	3.27	1.62-6.61	0.0009
	SSM	2.3	1.48-3.55	**0.0002**
rs1805761	Ulceration present	1.57	1.15-2.13	0.004
rs1028889	Extremity	1.73	1.16-2.59	0.007
rs6088520	Thickness 1–4 mm	0.61	0.46-0.80	**0.0004**
rs9960018	NM	1.91	1.29-2.84	0.001
rs12913832	NM	0.66	0.49-0.88	0.005
**SNP**	**Subgroup**	**Overall survival**		
		**HR**	**CI 95%**	***P***
rs12750212	Ulceration present	2.89	1.45-5.72	0.002
rs1805761	Ulceration present	1.72	1.17-2.55	0.006
rs9960018	NM	1.97	1.25-3.11	0.003
rs12913832	NM	0.66	0.45-0.97	0.03

Subgroups included tumor thickness (<1 mm, 1-4 mm, >4 mm), ulceration status (present/absent), anatomic site (axial/extremity), and histological type (SSM/NM). SSM = Superficial spreading melanoma, NM = Nodular melanoma. P-values that remain significant after Bonferroni correction (p < 0.0008; based on n = 64 independent tests, see Methods) are bolded. All subgroup associations were stratified by stage and adjustments included age, gender, ethnicity, anatomic site, tumor thickness, ulceration status, histological type, leaving out each dependent variable from the adjustment covariates in each respective subgroup analysis.

We have also performed logistic regression analyses between SNPs and particular clinical covariates (Table 5). Three highly correlated SNPs (r^2^ > 0.9) in the *PLA2G6* locus on chromosome 22q13.1 were significantly associated with primary tumor ulceration status, rs1028889 on chromosome 1p21.3 showed the strongest association with anatomic site, and rs966321 on chromosome 1p36.32 showed the strongest association with tumor thickness.

**Table 5:** Summary of SNP associations with ulceration status, anatomic site, and tumor thickness

**SNP**	**Locus**	**Gene**	**prior GWAS associations**		**Ulceration status**					
					**Crude**			**Adjusted**		
				**MAF**	**OR**	**CI 95%**	**P**	**OR**	**CI 95%**	**P**
rs6001027	22q13.1	PLA2G6	CM; OR = 0.83, p = 1.9e-8	0.35	0.71	0.53-0.92	0.01	0.70	0.53-0.92	0.01
rs2284063	22q13.1	PLA2G6	CM; OR = 0.83, p = 2.4e-9	0.35	0.73	0.56-0.96	0.02	0.73	0.55-0.95	0.02
rs132985	22q13.1	PLA2G6	Nevi; OR = 1.23, p = 2.6e-7	0.44	0.77	0.60-0.99	0.04	0.76	0.59-0.98	0.03
**SNP**	**Locus**	**Gene**	**prior GWAS Associations**		**Anatomic site**					
					**Crude**			**Adjusted**		
				**MAF**	**OR**	**CI 95%**	**P**	**OR**	**CI 95%**	**P**
rs7279297	21q22.3	PRDM15	Tanning; β = -0.12, p = 2.7e-6	0.31	1.26	1.01-1.56	0.03	1.32	1.05-1.66	0.01
rs1028889	1p21.3	-	Tanning; β = 0.10, p = 1.4e-4	0.27	0.81	0.65-0.99	0.04	0.74	0.58-0.92	0.008
rs6497287	15q13.1	HERC2	Eye color; p = 5.05e-15	0.12	0.70	0.53-0.93	0.01	0.70	0.51-0.93	0.01
rs7183877	15q13.1	HERC2	Hair; β = -.29, p = 2.0e-12	0.12	0.72	0.54-0.95	0.02	0.71	0.52-0.95	0.02
			Eye color; p = 6.18e-11							
**SNP**	**Locus**	**Gene**	**prior GWAS Associations**		**Thickness**					
					**Crude**			**Adjusted**		
				**MAF**	**β Coeff.**	**SE**	**P**	**β Coeff.**	**SE**	**P**
rs966321	1p36.32	-	Tanning; β = -.14, p = 1.6e-9	0.49	-0.40	0.14	0.005	-0.37	0.14	0.008
rs10861741	12q23.3	BTBD11	Hair; β = .12, p = 1.3e-4	0.15	-0.49	0.20	0.01	-0.47	0.19	0.01

SNP associations with ulceration status and anatomic site were observed using an additive model logistic regression analysis. SNP associations with primary tumor thickness were observed using an additive model linear regression analysis. Both crude (unadjusted by covariates) and adjusted results are shown; adjustments included age at diagnosis, gender, and ethnicity. Associations with melanoma and melanoma related host phenotypes observed in prior GWAS studies are also listed for each SNP.

**Figure 3:**
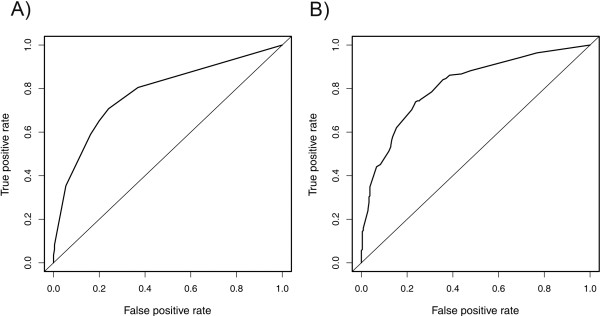
**ROC curves from logistic regression models for 3-year recurrence. A)** ROC curve from a model with stage and histological type as classifiers, AUC = 0.78. **B)** ROC curve from a model with stage, histological type, rs7538876, and rs9960018 as classifiers, AUC = 0.82.

Using multivariable logistic regression and ROC curves we have evaluated the top two SNPs associated with survival and recurrence (rs7538876, rs9960018) for their potential of improving the classification of recurred vs. non-recurred patients at 3-years follow up (N = 495; 252 recurred and 243 non-recurred) (Figure 3). In this analysis, additive models were assumed for both SNPs; rs7538876: p < 0.0001, OR = 2.14, 95% CI (1.52, 3.01); rs9960018: p = 0.002, OR = 1.74, 95% CI (1.09, 2.77). Including only stage and histological type as classifiers, the 3-year recurrence model has an AUC = 78%. With the addition of rs7538876 and rs9960018, the AUC significantly improves to 82% (p = 0.001, DeLong’s test), suggesting the potential role of both variants in prediction of patients at risk for recurrent disease.

**Figure 4:**
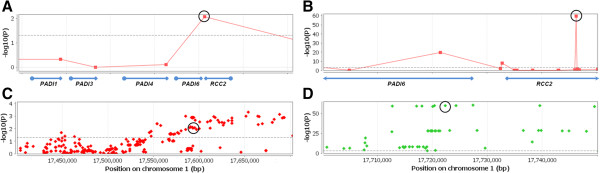
**eQTL and meQTL associations of rs7538876 with *RCC2*.** Association results for the eQTL analysis **(A and C)** and meQTL analysis **(B and D)** from 856 adipose tissue samples tested as part of the MuTHER project were generated by Illumina HT-12v3 Expression BeadChip, Illumina HumanMethylation 27 k array, and Illumina 610 k or 1 M chip. The expression and methylation levels of Illumina probes **(A and B)** associated with rs7538876 are plotted by –log10(p) (y-axis) vs. position on chromosome 1 (x-axis). The eQTL with the strongest association for rs7538876 (beta = 0.031, p = 0.009) was found for the probe ILMN_1720124 (circled) in *RCC2***(A)**. **C** shows the distribution of eQTL with SNPs across the *RCC2* region determined by the probe ILMN_1720124; circled is rs7538876. The strongest meQTL association for rs7538876 (beta = -0.042, p = 10^-60^) was found for cg07965774 (circled) in *RCC2***(B)**. The distribution of meQTL associations across the *RCC2* region determined by the cg07965774 probe indicates the presence of comparably associated meQTLs in highly correlated SNPs with rs7538876 (circled) **(D)**.

We have further tested whether rs7538876 affects the expression of *RCC2* in adipose tissue, in a similar pattern as described in the original BCC risk GWAS study [28]. We have used the data collected by the MuTHER project [25], a collaborative effort for the comprehensive assessment of disease association with expressed-quantitative trait loci (eQTL). In an eQTL analysis among adipose tissues from 428 female twin-pairs collected as part of MuTHER [25], we observed the strongest SNP-gene eQTL association for rs7538876 with *RCC2* (probe ID ILMN_1720124; p = 0.009) (Figure 4A, 4C). Increased expression was found to be associated with the minor allele [A] of rs7538876 (beta = 0.031), confirming the previous findings by Stacey *et al.*[28]. In addition, the MuTHER project also contains new data on the association of genetic variants with methylation status generated on the same set of adipose tissues from 428 twin-pair individuals. With this data available we were able to examine whether rs7538876 associates with the methylation in or around *RCC2*. We observed a highly significant association between rs7538876 and the methylation status of a CpG island within *RCC2* (probe ID cg07965774; p = 10^-60^), which was the strongest meQTL observed for this SNP (Figure 4B). Again, the association with minor allele [A] was correlated with decreased methylation in the *RCC2* locus (beta = -0.042). We have examined whether the meQTL effect of the same probe (cg07965774) replicates with other SNPs highly correlated with rs7538876 in this locus. We found the comparably significant meQTL associations as those observed for rs7538876 (Figure 4D), further supporting the validity of these findings.

We also tested putative functional impact of proxy SNPs in high linkage disequilibrium (r^2^ ≥ 0.9) with rs7538876. Using data from the Encyclopedia of DNA Elements (ENCODE) [27], we explored the putative regulatory roles of the proxy SNPs correlated with rs7538876 and found nine variants within transcription factor binding sites, six SNPs within DNaseI hypersensitivity clusters, and three SNPs within H3K4me1 chromatin marks (Table 6).

**Table 6:** **Putatively functional variants that correlate with our most significantly associated SNP, rs7538876, with an r**^**2**^ **> 0.9**

**SNP**	**Position**	**r2**	**Variant class**	**Gene**	**Putative function**
rs7538876	1:17594950	-	intronic	PADI6	-
rs12132197	1:17596551	1	intronic	PADI6	TFBS (STAT1, STAT2)
rs12132237	1:17596699	1	intronic	PADI6	DNase I hypersensitivity cluster
					TFBS (STAT1, STAT2)
rs7545115	1:17596918	1	intronic	PADI6	TFBS (STAT1, STAT2)
rs12134662	1:17597354	1	intronic	PADI6	TFBS (STAT1)
rs4920603	1:17599966	1	intronic	PADI6	DNase I hypersensitivity cluster
rs2526828	1:17602490	1	intergenic	-	DNase I hypersensitivity cluster
rs942457	1:17612173	1	exonic	RCC2	Synonymous
					TFBS (INI1)
rs1324367	1:17625038	0.903	intronic	RCC2	TFBS (HEY1)
rs11577822	1:17627195	0.935	intronic	RCC2	DNase I hypersensitivity cluster
					TFBS (HEY1)
rs1408420	1:17627402	0.935	intronic	RCC2	DNase I hypersensitivity cluster
					TFBS (HEY1)
rs4920607	1:17632685	0.935	intronic	RCC2	H3K4me1 mark
					TFBS (HEY1)
rs6586542	1:17636153	0.935	intronic	RCC2	H3K4me1 mark
					DNase I hypersensitivity cluster
rs6675912	1:17641877	0.903	intergenic	-	H3K4me1 mark

ENCODE database was used to establish transcription factor binding sites (TFBS), DNase I hypersensitivity clusters, and H3K4me1 chromatin marks. Variant annotation was performed using ANNOVAR.

## Discussion

We report for the first time the associations of melanoma-related GWAS risk loci with melanoma survival and other clinical outcomes. We also show that in addition to clinical variables, the incorporation of genetic information from our study into a logistic regression model significantly improves the classification of melanoma recurrence.

The high mortality rates associated with late stage melanoma and the emerging potential of new effective adjuvant therapeutics urge for the development of more personalized prognostic algorithms that complement the general clinical predictors. It is possible that the inherited genetic variants, associated with the risk of melanoma and host-related melanoma traits may serve as markers of disease prognosis; however, their prognostic potential has never been systematically investigated. Unlike most of the previous studies [11-17,29,30] which focused on a limited selection of genetic variants in candidate pathways, our scan has examined a comprehensive panel of 108 established genetic variants identified from recent GWAS on melanoma risk and melanoma host-related traits. Also, in contrast to many prior studies, the prospectively annotated population of more than 900 melanoma patients with detailed clinical information in our study allowed the assessment of both recurrence and overall survival, stratified by important clinicopathological characteristics.

In this study, we found the most significant association for rs7538876 with early recurrence, hence poorer outcome, in patients homozygous for the minor allele, recurring on average 2 years earlier compared to those carrying the major allele (multivariate HR = 2.41, p = 0.0002, Table 3). The association remained significant after Bonferroni correction (p = 0.01) and notably, the effect was consistent across different analyses (univariate, multivariable; Table 3) and multiple subgroup comparisons (tumor thickness, anatomic site, and histological type; Table 4), supporting a robust effect of this SNP on disease recurrence, regardless of other pathological characteristics. Interestingly, however, the effect was more pronounced in patients with non-ulcerated tumors and those with Breslow thickness <1 mm (Table 4), both clinical features of more favorable prognosis [5]. While this SNP was originally identified as a risk locus for basal cell carcinoma (BCC) [28], specifically for early-onset BCC, a risk for melanoma was not observed in the general melanoma population tested in this prior study. However, rs7538876 maps in 1p36, a locus frequently deleted in melanoma tumors and identified previously by linkage analysis in melanoma prone families [31,32], suggesting a possible, but yet unexplored genetic connection between BCC and familial melanoma risk in this region. To examine to what extent such interaction affects our findings we have tested whether our associations are confounded by the presence of cases with prior BCC history (n = 122), family history (FH) of melanoma (n = 139) or early-onset melanoma (<40 years of age) (n = 142) in our patient population. After adjusting the main effect analysis of melanoma recurrence for all patients separately by BCC prior history, FH status, and early onset at diagnosis, the overall association effect did not significantly change (p < 0.0003), indicating that these covariates do not contribute to our findings (data not shown). Interestingly, however, the associations were marginally, but consistently, significant in separate sub-analyses (separately testing the cases with prior history of BCC, FH, or early onset) providing an important cross-validation of our findings and a support for a general role of this SNP in melanoma recurrence, through a mechanism yet to be elucidated.

The SNP rs7538876 maps in the vicinity of Regulator of Chromosome Condensation 2 (*RCC2*), a gene involved in chromatin regulation during mitosis [33] and recently shown to be an essential regulator of cell cycle progression during interphase [34]. *RCC2* has also been shown to be involved in tumor invasiveness and metastasis, suggesting a putative role of this gene in melanoma progression [35-37]. Stacey *et al.* proposed the potential biological mechanism for this SNP through the up-regulation of *RCC2* by examining the expression data from adipose tissues and whole blood [38]. In the current study, due to the absence of RNA material from our population, we were not able to perform the expression analysis on melanoma specimens. Instead, we have examined the potential association of rs7538876 with the expression of *RCC2* in an independent set of adipose tissues collected as part of MuTHER project [25] (Figure 4A), confirming the association with expression found in the study by Stacey *et al.* (p = 0.009). More importantly, using the same adipose tissue resource, we found novel evidence suggesting that rs7538876 is strongly associated with CpG island methylation status within *RCC2* (p = 10^-60^) (Figure 4C). This presents a novel biological hypothesis suggesting that the alteration of *RCC2* expression by rs7538876 may be mediated through the epigenetic mechanism. The replication of the eQTL findings from a prior study [28] and the novel meQTL association of rs7538876 with *RCC2* found in our analysis, provide a highly promising rationale for the observed association effect with melanoma recurrence. In the most recent study, *RCC2* has been proposed to promote cell cycle progression [34]. The increased expression of *RCC2*, likely due to aberrant methylation, associated with “early recurrence” allele [A] in our study, adds further support for a putative oncogenic mechanism of this gene, possibly contributing to worse clinical outcomes and melanoma progression. While these suggestive links are intriguing, the follow-up molecular analyses on tumors and normal tissues from melanoma patients will be needed to confirm these findings.

We also explored other potential mechanisms by which rs7538876 may modulate melanoma recurrence. It is possible that rs7538876 may only be a surrogate for other variants highly correlated with rs7538876, but with strong functional impact. As illustrated in Table 6, we identified several variants within transcription factor binding sites or DNaseI hypersensitivity loci, which may also potentially affect the expression of other nearby or possibly distant genes (in cis or trans configuration). Interestingly, rs7538876 and several other correlated variants map within the *PADI6*, which is involved in cytoskeletal organization [39], but due to its expression in early embryogenesis, its role in melanoma progression is yet to be elucidated. As part of future studies, the detailed fine mapping and eQTL analysis in melanoma tissues will be needed to further refine the association effect with melanoma recurrence driven by rs7538876.

A second strong locus associated with early recurrence and more significantly with reduced overall survival in our study is rs9960018. This SNP, originally associated with reduced tanning response in a recent GWAS [40], maps in *DLGAP1,* a gene involved in pathways often dysregulated in malignant melanoma including cell migration, the extracellular matrix and cytoskeleton networks [41], Interestingly, *RCC2* (rs7538876) and *DLGAP1* (rs9960018) are both involved in integrin signaling, which is frequently altered in metastatic melanoma [41], suggesting a possible molecular interplay in melanoma progression. Pending an experimental validation, the notion of common functional pathways involving both loci may provide further support for the observed associations of rs7538876 and rs9960018 with disease outcomes.

Both rs7538876 and rs9960018 were also associated with survival in subset analyses, among superficially spreading melanoma (SSM) and nodular melanoma (NM), respectively (Table 4). Interestingly, in these analyses we found the preferential association of rs7538876 with earlier recurrence in SSM but not NM, and conversely the strong survival effect (both on OS and RFS) for rs9960018 in NM but not SSM. Because these subtypes are characterized by different clinical presentations, it has been debated whether they are consequential events of melanoma progression or independent clinical entities. Several studies by our group and others have supported different molecular characteristics of NM versus SSM that cannot be reconciled by the linear progression model [42-45]. The specific association effects observed with melanoma outcomes for rs7538876 and rs9960018 in SSM and NM, respectively, give further support that SSM and NM are two distinct clinical and prognostic entities requiring separate prognostic assessment.

Our findings demonstrate the potential importance of assessing melanoma prognosis by combining clinicopathological characteristics with genetic information. Using a logistic regression model, we show that the incorporation of rs7538876 and rs9960018 significantly improves the classifier of 3-year recurrence compared to stage and histological type alone (AUC = 82% versus AUC = 78%, respectively, p = 0.001). This not only supports the prognostic impact of associations identified here but it also outlines the practical utility of these findings for downstream clinical applications. Specifically, the association of rs7538876 with worse outcome in patients with otherwise favorable clinical characteristics (thin and non-ulcerated melanomas, Table 4), illustrates the potential power of genetic information to identify high-risk patients from otherwise low-risk subsets, hence providing more refined prognostic information in addition to melanoma AJCC clinical variables.

One possible concern in the current study may be the lack of host phenotype information for the patients, as some melanoma host phenotypes (e.g. pigmentation) have been suggested to modify disease risk [46-48], and also affect survival [49-51]. In our data, this can be the case for two variants associated with recurrence; SNPs in the “pigmentation” locus of *MC1R* and rs12913832, a variant originally associated with blue eye color (for the major allele) [52], which in our study shows correlation with more favorable outcome for the minor allele (darker pigmentation). Although these associations are marginal, the availability of phenotype data as part of a larger validation, e.g. in a population tested recently [12], may provide a more complex assessment of host factors potentially impacting the observed associations.

While the findings presented here warrant validation in an independent population, our study employs one of the largest melanoma prospective subsets ascertained to date from a single center. In a recent study by Davies *et al.*[12], the authors stress a need for large consortia in melanoma prognostic assessment of common genetic variants. Although such a strategy is indeed relevant for replication purposes, the multicenter “discovery” meta-analysis in the context of clinical outcome can be hampered by numerous biases, as also noted in that prior report [12], and discussed extensively in many other previous studies [53-56]. These biases may include the inter-study differences in patient enrollment, clinical procedures, follow-up data uniformity, and patient characteristics (e.g. geographical and host-exposure differences). In contrast, our analysis employs a population followed up from the time at diagnosis at a single institution and managed under standardized criteria for diagnosis and treatment, hence reducing the expected clinical heterogeneity of end-point estimates.

Another potential limitation may relate to the drug intervention, which was not accounted for in our analysis. In particular, adjuvant therapy (AT) can provide a specific survival benefit of later recurrence, mainly for stage III melanoma patients [57]. Because only a small subset of patients in our study was treated with AT this will unlikely impact the overall findings. To test this possibility we have performed a separate comparison including only stage I and II patients and saw the results did not change significantly, suggesting that the presence of advanced stages previously treated with AT does not impact our analysis.

In summary, the comprehensive assessment of germline variants associated with melanoma risk or host-related phenotypes from prior GWAS in our study shows for the first time that the germline risk loci may impact melanoma outcomes. These novel findings are highly promising and strongly support the need for further independent validation. This is particularly important for the results of the sub-analyses, where the power reduction of sample size may be a concern. While independent analysis will be needed to add additional support to our conclusions, the promising associations of common genetic risk variants with melanoma outcomes found here not only propose clinical implications, but also suggests for the first time that germline genetic variation may have a broader role in melanoma progression. In this context it will also be important as part of a subsequent replication analysis, to further the discovery of additional prognostic germline genetic loci, including those that are unrelated to melanoma risk, but are involved in important pathways in melanoma progression. Such separate prognostic scans, possibly on a genome-wide level, will likely be highly beneficial not only for the identification of additional prognostic biomarkers, but also for the discovery of novel pathways involved in melanoma progression revealing potential targets for more efficient treatment strategies.

## Conclusions

Germline genetic variants previously identified in GWAS as risk loci for melanoma and melanoma host-related phenotypes showed association effects on melanoma recurrence-free and overall survival. In particular, the most significant associations were found for rs7538876 with early recurrence and rs9960018 with both early recurrence as well as overall survival. When incorporated into a logistic regression model with other clinicopathological characteristics, these two SNPs showed a significant improvement in classification of 3-year melanoma recurrence. This evidence suggests that the germline genetic variants associated with melanoma risk may also modulate melanoma prognosis.

## Competing interests

The authors declare that they have no competing interests.

## Authors’ contributions

The study concept and design were devised by IO and TK. Data generation was performed by JR, JS, CD, AD, MM, IO, and TK. JR, SS, YS, and TK contributed to the data analysis and interpretation. JR, SS, CD, CA, PS, YS, and TK participated in drafting the manuscript. RS, RB, AP, DP, YS, IO, and TK revised the manuscript for critical intellectual input. Funding was obtained by IO and TK. Administrative, technical, or material support was provided by RS, RB, AP, DP, IO, and TK. TK supervised the study. All authors read and approved the final manuscript.

## Supplementary Material

Additional file 1 Reference studies for SNP selections. Click here for file
